# Assessing the Impact of Sample Heterogeneity on Transcriptome Analysis of Human Diseases Using MDP Webtool

**DOI:** 10.3389/fgene.2019.00971

**Published:** 2019-10-24

**Authors:** André N. A. Gonçalves, Melissa Lever, Pedro S. T. Russo, Bruno Gomes-Correia, Alysson H. Urbanski, Gabriele Pollara, Mahdad Noursadeghi, Vinicius Maracaja-Coutinho, Helder I. Nakaya

**Affiliations:** ^1^Department of Clinical and Toxicological Analyses, School of Pharmaceutical Sciences, University of São Paulo, São Paulo, Brazil; ^2^Advanced Center for Chronic Diseases–ACCDiS, Facultad de Ciencias Químicas y Farmacéuticas, Universidad de Chile, Santiago, Chile; ^3^Division of Infection and Immunity, University College London, London, United Kingdom; ^4^Scientific Platform Pasteur–USP, São Paulo, Brazil

**Keywords:** heterogeneity, transcriptome analysis, gene expression profiling, infectious diseases, inflammatory diseases

## Abstract

Transcriptome analyses have increased our understanding of the molecular mechanisms underlying human diseases. Most approaches aim to identify significant genes by comparing their expression values between healthy subjects and a group of patients with a certain disease. Given that studies normally contain few samples, the heterogeneity among individuals caused by environmental factors or undetected illnesses can impact gene expression analyses. We present a systematic analysis of sample heterogeneity in a variety of gene expression studies relating to inflammatory and infectious diseases and show that novel immunological insights may arise once heterogeneity is addressed. The perturbation score of samples is quantified using nonperturbed subjects (i.e., healthy subjects) as a reference group. Such a score allows us to detect outlying samples and subgroups of diseased patients and even assess the molecular perturbation of single cells infected with viruses. We also show how removal of outlying samples can improve the “signal” of the disease and impact detection of differentially expressed genes. The method is made available *via* the mdp Bioconductor R package and as a user-friendly webtool, webMDP, available at http://mdp.sysbio.tools.

## Introduction

Gene expression profiling methods such as microarrays and RNA-seq have been extensively used to examine the molecular changes associated with a biological “perturbation.” This perturbation can be drug treatments, vaccinations, infections, cancers, and autoimmune or inflammatory diseases (Nakaya et al., 2012; Prada-Medina et al., 2017; Jochems et al., 2018). For human diseases, the initial analysis usually tries to find genes whose expression is significantly altered in the perturbed condition (i.e., patients with the disease) compared to the nonperturbed subjects (i.e., the healthy subjects). However, the definition of health and disease is broad, and the inherent variation among individuals can make any group of human samples highly heterogeneous. Variation can be due to genetic and environmental factors, as well as undetected health problems (Whitney et al., 2003; Albert and Kruglyak, 2015). Similarly, patients with the same disease can present huge variation in terms of symptoms or score (Hersh and Prahalad, 2015; Garg and Smith, 2015). Thus, the removal of outlier samples can impact downstream analyses, especially in studies investigating mild diseases or the administration of inactivated vaccines.

Transcriptome datasets typically contain expression values of tens of thousands of genes from a relatively small number of samples. This presents a dimensionality problem when trying to identify significant changes in gene expression (Wang et al., 2008). Most methods will classify a gene as differentially expressed if there is a large difference in the mean expression between classes and a low variance within classes (De Hertogh et al., 2010). Therefore, genes that have heterogeneous expression within a class due to technical or biological outliers will have their detection as differentially expressed hindered. Individual heterogeneity can arise from past infections, environmental factors, microbiota, and genetics (Gibson, 2008), as well as undetected problems such as chronic disease, worms, food poisoning, or asymptomatic infection. In order to reduce biological heterogeneity, scientists try to enroll subjects with similar characteristics, controlling them for gender, clinical information, age, and so on. However, many hidden factors will invariably remain in the final set of samples and contribute to individual differences.

The molecular distance to health (Pankla et al., 2009) is a method that analyzes sample heterogeneity by scoring samples based on how distant their expression is to healthy and has been applied to quantify the perturbation of samples from diseased subjects (Berry et al., 2010; Banchereau et al., 2012; Bell et al., 2016). However, there has been no systematic assessment of how human heterogeneity affects downstream analyses. Also, none of the previous studies have used specific knowledge-based gene sets to evaluate subject perturbation or provided a tool for users to assess the heterogeneity in their own datasets.

Here we describe a systematic analysis on heterogeneity of several RNA-seq and microarray datasets from a diverse set of human diseases. Our approach, called the molecular degree of perturbation (MDP), is available as a Bioconductor R package (https://bioconductor.org/packages/release/bioc/html/mdp.html) and can identify potentially problematic subject data from transcriptomic dataset, as well as to quantify the perturbation score of healthy and diseased samples. Meanwhile, our user-friendly web-based application (https://mdp.sysbio.tools/) allows scientists to run MDP without any knowledge of bioinformatics or programming languages. We demonstrated that the application of our method on inflammatory and infectious disease datasets can affect the detection of differentially expressed genes (DEGs). Finally, these tools were used to analyze RNA-seq data of single cells infected with dengue virus (DENV), revealing the individual cell heterogeneity of infected cells.

## Methods

### MDP Algorithm

The MDP score measures how much a sample is distant from a reference group of samples. Let *G* be the genes in a given expression dataset with *N* samples, out of which *h* are the healthy control samples. Also, let be a centrality measurement (either the mean or the median; the default is median), and , a measure of the variability (the standard deviation or the MAD) for each gene *i* in the control samples. Finally, let *z* *_i_* be a modified *z*-score transformation using and as parameters. The absolute values of *z* *_i_* are taken, and values less than 2 are set to 0. The values that remain represent significant deviations from the healthy control samples. The MDP score for each sample *j* (both in the control and perturbed groups) is then the mean of the modified absolute *z* *_i_* values considering all genes or just the perturbed ones. The “perturbed genes” represent the top (default is 25%) genes with the highest absolute *z* *_i_* values across all samples in a perturbed group. Additionally, the MDP package can automatically identify outlier samples based on the number of standard deviations (default = 2) from the mean of MDP scores of all samples within each class.

### Data Acquisition and Processing

Normalized gene expression data from RNA-seq and microarray studies were downloaded from the GEO database (https://www.ncbi.nlm.nih.gov/geo/). If normalized data were not available, we processed the raw CEL files using the affy Bioconductor R package (Gautier et al., 2004) and performed data quality control using the arrayQualityMetrics Bioconductor R package (Kauffmann et al., 2009). Normalization was performed using the “RMA” function from the affy package. Samples that failed at least two quality control tests before or after normalization were removed from downstream analyses. For the single-cell RNA-seq data, we utilized the gene counts table from Supplementary File 7 published by Zanini et al. (2018). Prior to the calculation of MDP on single-cell data, we kept only the top 30% genes with the highest mean expression on all single cells and then removed the genes with zero values in 40% or more single cells.

### Differential Gene Expression Analysis

Student *t* test was used to identify DEGs between patients with a disease and the healthy subjects. Different log_2_ fold change and adjusted *P* value (Benjamini and Hochberg) cutoffs were used and are shown in **Table S1**.

### Pathway and Network Analyses

We used the NetworkAnalyst tool (Xia et al., 2015) to create the protein–protein interaction network with the DEGs. For the JIA analysis, we used the protein–protein interaction database STRING (score >900) and the minimum network. For the single-cell RNA-seq analysis, we used the protein–protein interaction database STRING (score >900) and the zero-order network. Overrepresentation analyses using the Gene Ontology gene sets were performed using the genes in the networks. Cytoscape software (Shannon et al., 2003) was used to display the networks.

### MDP Webtool Implementation

The code of the tool was implemented in HTML, CSS, JavaScript, PHP, and R. To upload files, check for errors and check the structure of the data; we used the languages JavaScript and PHP. An R script containing the https://cloud.r-project.org repo packages: data.table, withr, ggplot2, plotly, and pandoc was used to process the data and generate the results in HTML.

For defining style and appearance of pages, we used CSS with Bootstrap, which is a front-end framework with several components included. For dynamic manipulation of the page, we used JavaScript with Jquery. The latter is a framework for JavaScript itself, where its main purpose is to facilitate, streamline, and reduce the complexity in development.

In the infrastructure, we used the concept of containers and microservice with the platform Docker. In parallel, we used the tool Docker Compose to orchestrate and to deploy these containers. In total, we have three containers: proxy, nginx, and php-fpm. In the proxy container, the functions of reverse proxy and load balancing were performed, which were left in charge of the traefik service (https://traefik.io/). It also implements SSL certificate management through the Let’s Encrypt project (https://letsencrypt.org/). The nginx container is our webserver, and the php-fpm is the backend that processes requests to php files.

## Results

### Molecular Degree of Perturbation Algorithm and Webtool

**Figure 1:**
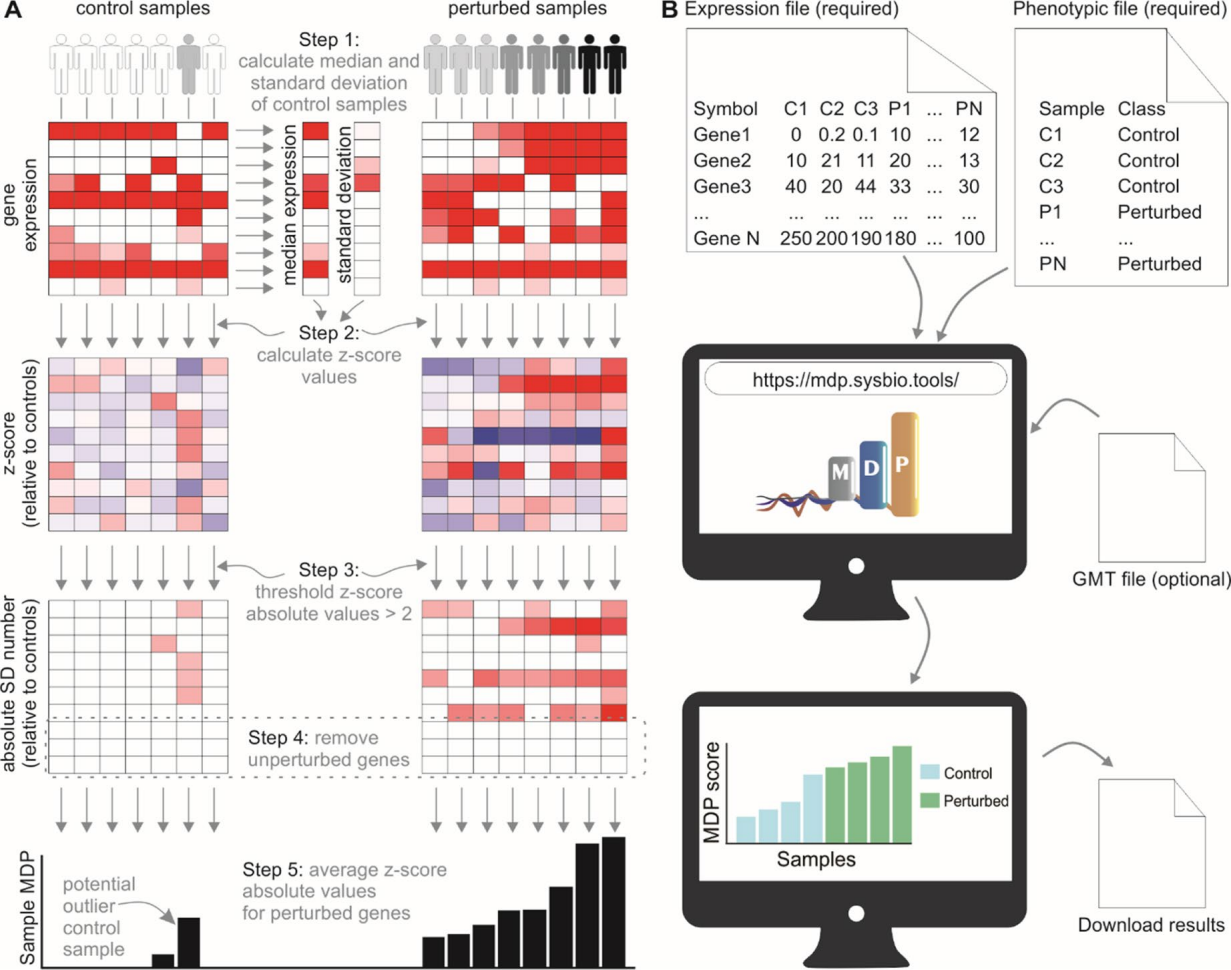
The molecular degree of perturbation approach to calculating sample heterogeneity. **(A)** The MDP algorithm scores samples based on their perturbation from user-defined control samples (often healthy subjects). A *Z*-score normalization is performed using the control samples as a reference. The absolute values of the normalized scores are then taken, and values below 2 are set to 0. The sample scores are the average of these gene scores for each sample. **(B)** Running the MDP webtool. Expression and phenotypic files are required to run MDP; the results are a simple barplot and boxplot showing molecular perturbation for each submitted sample. An optional feature allows users to run MDP using a specific gene set, provided as a.gmt file.

We developed a user-friendly tool that inspects sample heterogeneity by assigning a score to each sample based on the cumulative perturbation of its gene expression levels relative to control samples. The algorithm performs a *Z*-score normalization of gene expression values for noncontrol samples, using the control samples to compute the median (M) and median absolute deviation (MAD). Absolute normalized expression values less than 2 are designated as unperturbed and are set to 0. Sample MDP scores are the average of normalized expression values for a given gene set (**Figure 1A**).

The web interface for MDP (http://mdp.sysbio.tools) has been developed to allow non–bioinformatics users to quickly assess the MDP in their samples without the need for any previous computational knowledge or additional software (**Figure 1B**). The minimal requirements to execute the webtool are the input gene expression file and the phenotype data file. As long as the data are already normalized (CPM, TMM, FPKM, RMA, etc.), gene expression data from both RNA-seq and microarray experiments are supported.

The MDP tool has an additional feature that allows users to assess the MDP using a specific gene set or pathway. This may be useful in cases where there is a prior knowledge about the pathways involved with the disease. For running this optional analysis, users must provide a pathway annotation file in.gmt format and then select a specific gene set or pathway to calculate the perturbation score.

### The Sample Perturbation Score for Different Human Diseases

We applied the MDP to 20 transcriptome studies (11 microarray and 9 RNA-seq) obtained from the GEO (Edgar et al., 2002) and SRA (Leinonen et al., 2011) databases in order to investigate how sample heterogeneity can impact the downstream differential expression analysis. Studies were related to tuberculosis (TB), cancer, juvenile idiopathic arthritis (JIA), sepsis, and other autoimmune and infectious diseases.

We initially showed that the perturbation scores of samples broadly vary within and between different diseases or treatments (**Figure S1**). Infection with the bacteria *Staphylococcus aureus*, for instance, seems to be a stronger perturbation than infection with influenza virus (**Figure S1A**) (Ramilo et al., 2007). Similarly, different types of cancer may show lower or higher perturbation scores regardless of their known prognostic values (**Figure S1B**) (Best et al., 2015). Our approach also differentiates between several subtypes of inflammatory diseases such as JIA, Crohn disease, and ulcerative colitis (**Figure S1C**) (Mo et al., 2018).

### MDP Identifies Potential Outlier Samples

**Figure 2:**
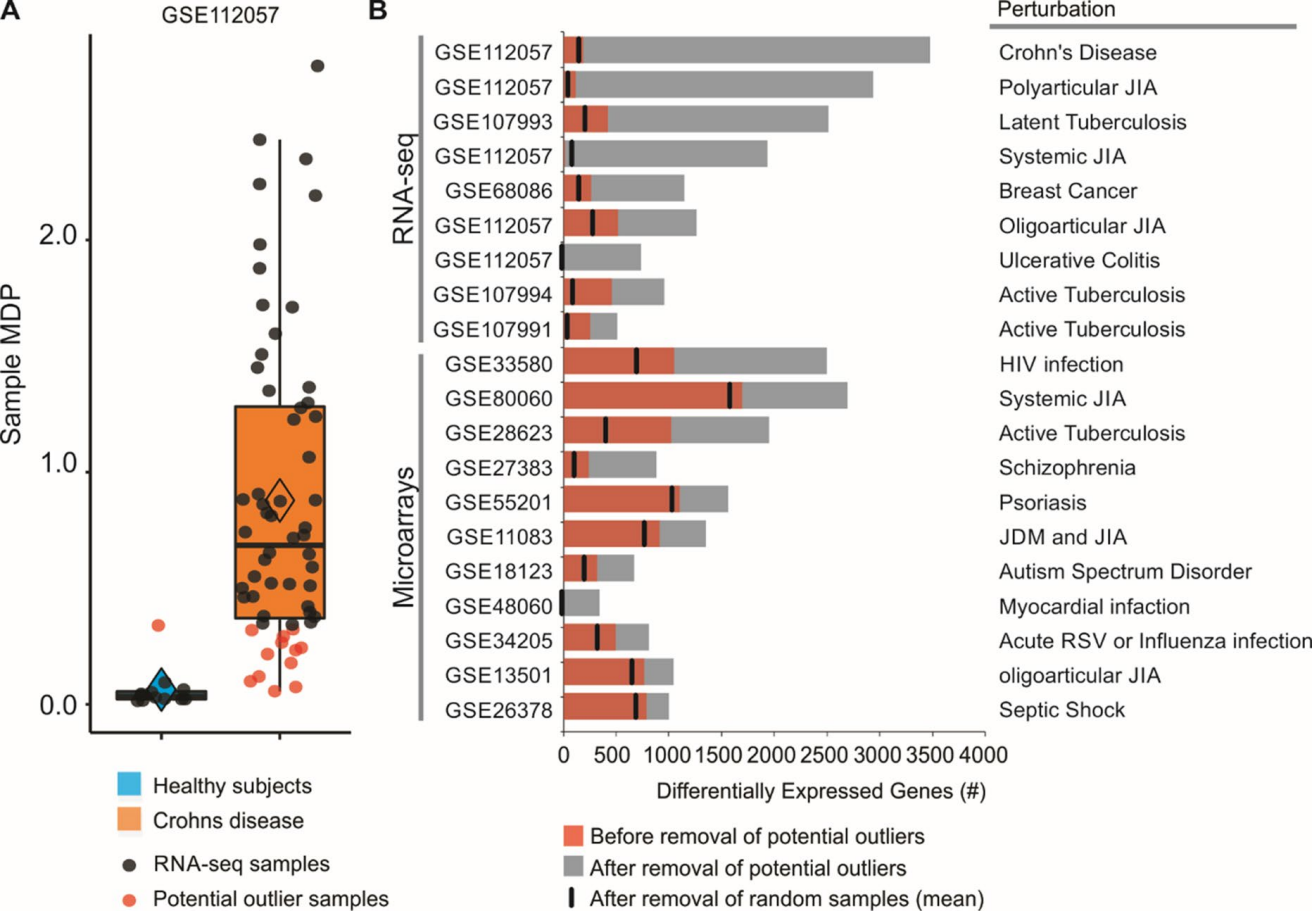
Removal of potential outlier samples impacts differential expression analyses. **(A)** Sample MDP scores were calculated for 60 patients with Crohn disease using as a reference group 12 healthy subjects. Data were obtained from whole blood and are available under GEO accession GSE112057. Healthy subjects (blue) were used as reference group. Potential outlier samples are shown as red dots. **(B)** Differential expression analyses between patients with a disease and healthy controls. Numbers of DEGs before and after removal of potential outlier samples are shown as red and black bars, respectively. Random removal of samples followed by differential expression analysis was performed 1,000 times for each comparison, and the number of DEGs was averaged (black vertical line).

By assessing the sample perturbation scores, we were able to identify potential outlier samples for each of the 20 microarray and RNA-seq studies. One representative boxplot (**Figure 2A**) shows that one of the healthy subjects may be in fact “perturbed” when compared to the rest of the healthy group. Similarly, 12 of Crohn disease patients do not seem greatly perturbed at the molecular level (**Figure 2A**). Treating these samples as outliers and thus removing them from differential expression analyses increased the number of DEGs. For the GSE112057 comparison between healthy subjects and Crohn disease patients, we identified 188 DEGs before the removal of outliers (**Figure 2B**). After removal, the number of DEGs for this comparison was 3,477 (18.50-fold increase). If only the single control outlier sample is removed (**Figure 2B**), the number of DEGs increases to 1,931 (10.1-fold increase). We also randomly removed the same number of samples considered as outliers and counted the number of DEGs for each comparison. This process was repeated 1,000 times showing that the increase in DEG number is not due to random chance (**Figure 2B**). We performed this analysis for the 19 other comparisons as well. In all of them, the number of DEGs increased after removing the potential outliers (**Figure 2B**).

### Removal of Potential Outlier Samples Increases Biological Consistency Across Similar Studies

**Figure 3:**
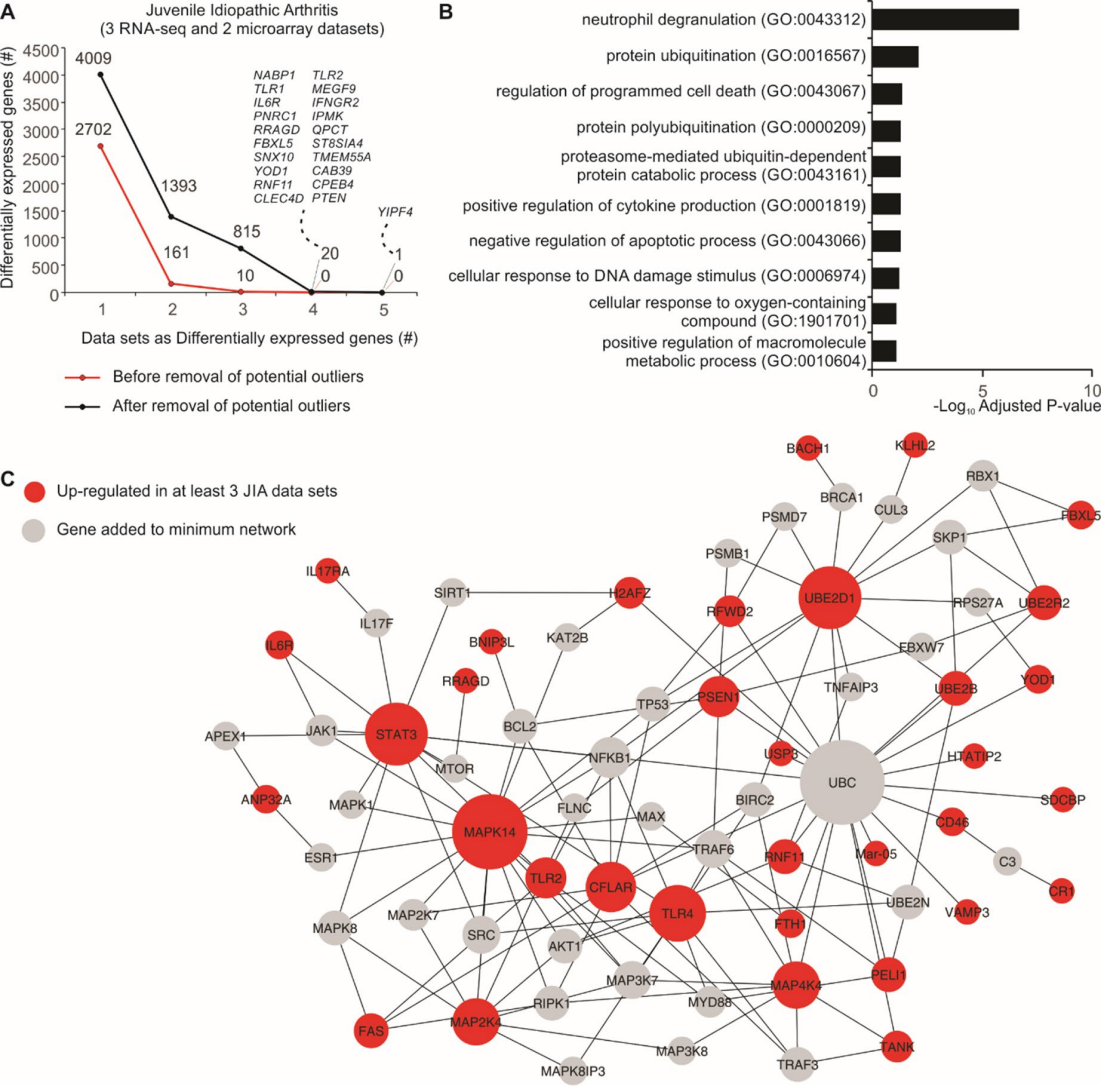
Consistency of JIA signatures increases after removal of potential outlier samples. **(A)** Number of DEGs before and after removal of potential outlier samples in five JIA datasets. The lines show the number of genes (*y* axis) considered as DEGs in one or more JIA datasets (*x* axis). **(B)** Enrichment pathway analysis of genes consistently up- or down-regulated in three or more JIA datasets after removal of potential outlier samples. Bar graph shows the log_10_ adjusted *P* value (*x* axis) of top Gene Ontology gene sets (*y* axis). **(C)** Protein–protein interaction network showing the connectivity of up-regulated DEGs in at least three JIA datasets. Genes added to minimum network are shown as gray nodes. Edges were defined by InnateDB (Breuer et al., 2013).

Five JIA datasets (three RNA-seq and two microarrays) were used to assess the consistency between DEGs before and after removal of potential outlier samples identified by MDP. After removal, we found 21 genes that were differentially expressed in at least four JIA datasets, and none using all original samples (**Figure 3A**). Overrepresentation analysis of the genes consistently up-regulated in three or more datasets revealed that the top 1 gene set, neutrophil degranulation (GO:0043312), was recently associated with JIA (Brown et al., 2018) (**Figure 3B**). We then created a protein–protein interaction network with these consistently up-regulated genes (**Figure 3C**). This approach revealed highly connected genes, which may be central to JIA, such as STAT3, UBE2D1, MAPK14, and TLR4 (**Figure 3C**).

### Using a Specific Gene set to Determine the MDP

T cells play a critical role in the outcome of *Mycobacterium tuberculosis* infection (Jasenosky et al., 2015). One important cytokine released by these cells is interferon gamma (IFNg). However, Berry et al. (2010) have shown that the blood transcriptome of patients with active TB was dominated by neutrophil-driven type I IFN-related genes. We thus decided to evaluate if gene modules related to specific blood immune cell populations can capture the MDP of patients with active TB. In the analysis, we used transcriptional modules that have been extensively validated to be highly specific for different immune cell types (Pollara et al., 2017). We also used modules derived from the unique transcriptome of human monocyte-derived macrophages (Mф) stimulated *in vitro* with different cytokines (Bell et al., 2016). For the study GSE19435 (Berry et al., 2010), the sample MDP scores calculated with gene modules of macrophages treated with IFNg for 4 h, neutrophils and T cells were higher in patients with active TB compared to those from healthy controls (**Figure S2A**). We also performed the same analysis for all 15 gene modules and all 7 TB datasets (**Figure S2B**) and found that the genes associated with macrophages treated with IFNg for 4 or 24 h are greatly perturbed in active TB. This analysis demonstrated that prior knowledge about a disease can be used to quantify sample perturbation and that the gene set used will impact the MDP scores.

### MDP Analysis for Single-Cell RNA-Seq Dataset

**Figure 4:**
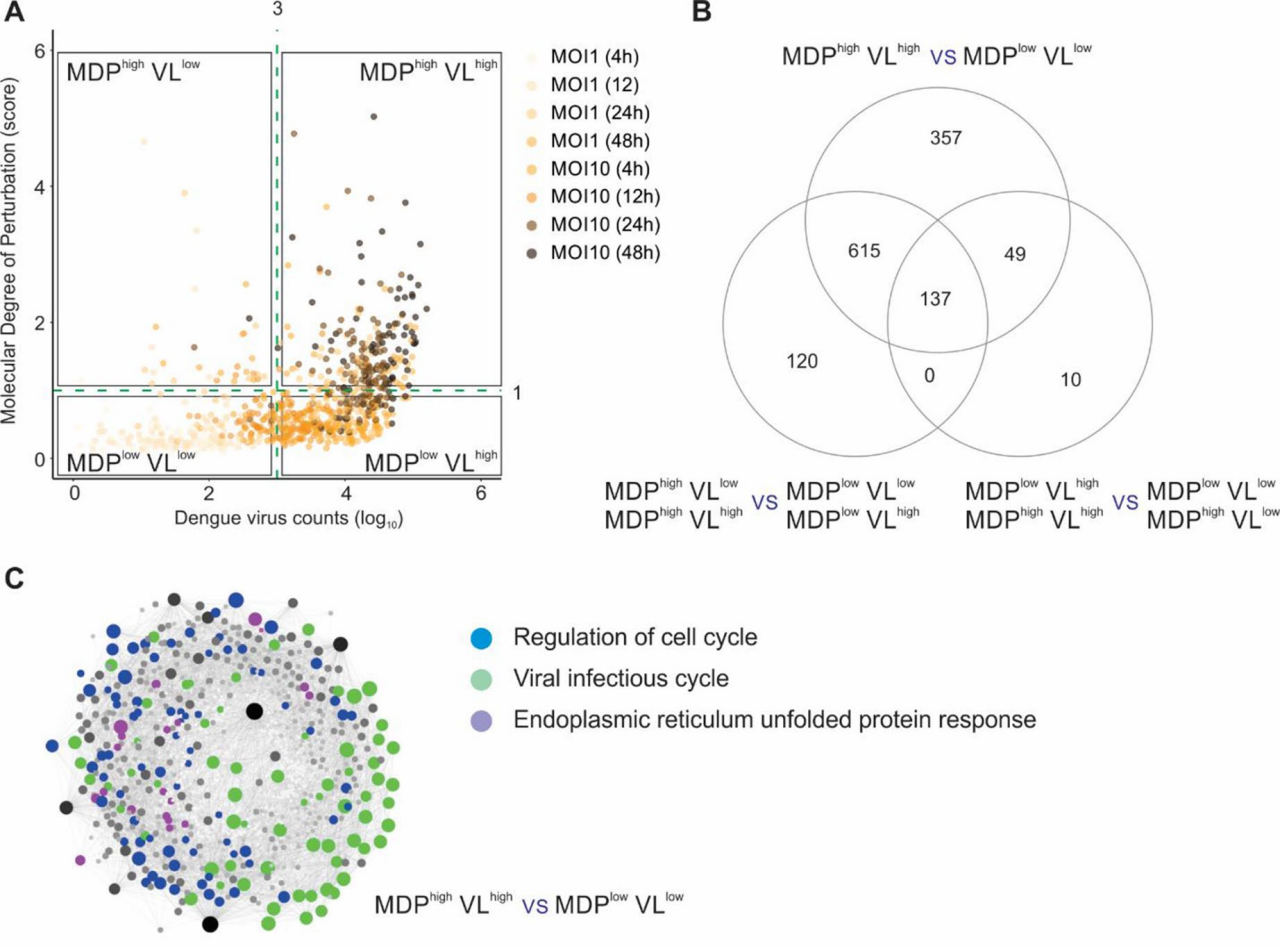
Molecular degree of perturbation at single-cell level. **(A)** Sample MDP score (*y* axis) and DENV count (VL, *x* axis) for all single cells infected with DENV. The quadrants divide cells according to their MDP score (cutoff = 1) and/or the VL (cutoff = 3). Each circle represents a single cell, and the gradient color represents the different MOI (*m*ultiplicity *o*f *i*nfection) and time postinfection. **(B)** Differential expression analyses between cell subsets shown in A. The numbers of DEGs are indicated in the Venn diagram. **(C)** Protein–protein interaction network using the DEGs from MDP^high^VL^high^ versus MDP^low^VL^low^ comparison. Genes related to regulation of cell cycle (blue circles), viral infectious cycle (green circles), and endoplasmic reticulum unfolded protein response (purple circles) are shown.

Finally, we applied the MDP approach to analyze the molecular perturbation caused by a viral infection at single-cell level. Zanini et al. (2018) developed an approach named viscRNA-seq (virus-inclusive single-cell RNA-seq) to probe the host single-cell transcriptome together with intracellular viral RNA. We first evaluated if the MDP score was correlated with the DENV counts (herein defined as viral load or VL). Using uninfected single cells as the reference control, we calculated the MDP score for all cells infected with DENV and then compared these scores with VL (**Figure 4A**). No clear correlation was seen between MDP score and VL. Based on the VL (cutoff VL = 10^3^) and on the MDP score (cutoff MDP = 1), we split the single cells into four subsets: MDP^high^VL^low^, MDP^high^VL^high^, MDP^low^VL^low^, and MDPl^ow^VL^high^. We then performed differential expression analyses between these subsets to assess the transcriptomic alterations caused by DENV infection. **Figure 4B** shows that the highest number of DEGs was found when we compared MDP^high^VL^high^ with MDP^low^VL^low^ subsets (1,158 DEGs), rather than either of these criteria alone. Comparing cells with high MDP score (MDP^high^VL^low^ + MDP^high^VL^high^) with those with low MDP score (MDP^low^VL^low^ + MDPl^ow^VL^high^) resulted in 872 DEGs. The lowest number of DEGs (196 DEGs) was found when we compared cells with high VL (MDP^high^VL^high^ + MDP^low^VL^high^) with those with low VL (MDP^high^VL^low^ + MDPl^ow^VL^low^) (**Figure 4B**). These results suggest that VL alone cannot be a strong marker of cell perturbation.

Network and pathway analyses were then performed on the 1,158 DEGs identified in the MDP^high^VL^high^ with MDP^low^VL^low^ comparison (**Figure 4C**). The top associated pathways were “regulation of cell cycle,” “viral infectious cycle,” and “endoplasmic reticulum unfolded protein response” (**Figure 4C**). In addition to VL, MDP provided another layer of information for quantifying heterogeneity at single-cell level and generated novel insights associated to viral infections.

## Discussion

We have shown that the MDP tool provides an intuitive way to inspect gene expression data and identify samples that are potential biological outliers. Although it can be argued that it is important to embrace the heterogeneity of samples and use all of them to perform analyses, we have shown that, for DEG analyses, sample removal can result in a dramatic improvement in the number of DEGs found, particularly removal of clear outlier samples in an otherwise uniform control group. Removing perturbed outliers could also potentially prove useful for finding disease classifiers by increasing the consistency of DEGs between similar studies. For single-cell analyses, it is not clear, however, how dropouts and cells with low MDP scores may impact the interpretation of the results since zero-inflated datasets may affect the calculation of MDP.

We observe that there is a great variation in the transcriptional profile of patients with different diseases. Part of this variability is due to the genetic contributions of each individual, as well as their prior infections, nutritional condition, stress, microbiota, and so on (Nakaya et al., 2012). There is still the possibility of hidden comorbidities in the diseased individuals, which were not part of the exclusion criteria of the clinical trials. The degree of molecular perturbation can provide a good indication of the health status of the individual and also identify the genes most perturbed by the disease in question.

Finally, the MDP approach can also be used to identify disease-associated perturbation in a priori–defined clinical or immunological factors (Bell et al., 2016; Pollara et al., 2017). In this way, the analysis can be used to split patients with the same disease into new subgroups with distinct gene expression profiles.

## Data Availability Statement

Publicly available datasets were analyzed in this study. These data can be found here: https://www.ncbi.nlm.nih.gov/geo/.

## Author Contributions

AG, ML, and HN performed the analyses, wrote the initial draft, and developed the tools. PR, AU, GP, and MN performed analyses. BG-C and VM-C implemented and help developed the webtool version. HN supervised the work. All authors wrote the final version of the manuscript.

## Funding

This work was supported by grants from FAPESP (2012/19278-6, 2013/08216-2, 2018/14933-2), CNPq (313662/2017-7), FONDECYT-CONICYT (11161020), and PAI-CONICYT (PAI79170021). This study was financed in part by the Coordenação de Aperfeiçoamento de Pessoal de Nível Superior–Brasil (CAPES)–Finance Code 001. MN and GP were supported by the Wellcome Trust and National Institute for Health Research Biomedical Research Centre at University College London Hospitals.

## Conflict of Interest

The authors declare that the research was conducted in the absence of any commercial or financial relationships that could be construed as a potential conflict of interest.

## Supplementary Material

Figure S1 Sample MDP scores of different human diseases. **(A)** Sample MDP scores of patients acutely infected with either virus or bacteria. Data were obtained from blood leukocytes and are available under GEO accession GSE6269. Healthy subjects (blue) were used as reference group. **(B)** Sample MDP scores of different types of cancer. Data were obtained from platelets and are available under GEO accession GSE68086. Healthy subjects (blue) were used as reference group. **(C)** Sample MDP scores of patients with inflammatory diseases. Data were obtained from whole blood and are available under GEO accession GSE112057. Healthy subjects (blue) were used as reference group.

Figure S2 MDP calculated with specific gene modules. **(A)** Sample MDP score of patients with active TB (brown bars) and healthy controls (blue bars) using three different specific gene modules. Data were obtained from whole blood and are available under GEO accession GSE19435. **(B)** Sample MDP score calculated using all gene modules and for all TB datasets. The circles represent the difference between the median sample MDP score of patients with active TB and the healthy controls with no active TB within each study. The size and color of the circles are proportional to this difference. MФ: macrophages.

Table S1 Differential expression analysis with or without removal of potential sample outliers. The transcriptomic studies are shown as rows. StudyId = number of the study; GEOId = GEO accession ID with the type of disease; TotalControlSamples = number of samples in control group; TotalTreatedSamples = number of samples in disease group; TotalControlOutliers = number of samples in control group that were considered outlier by MDP; TotalTreatedOutliers = number of samples in disease group that were considered outlier by MDP; TotalOutliers = number of samples in total that were considered outlier by MDP; DEGsBefore = number of differentially expressed genes without removing any potential sample outlier (using samples in TotalControlSamples and TotalTreatedSamples); DEGsAfter = number of differentially expressed genes after removing potential sample outliers (using samples in TotalControlOutliers and TotalTreatedOutliers); DEGMin = minimum number of differentially expressed genes found after removing random samples (number of samples removed on each itineration is equivalent to the corresponding number in TotalOutliers) from TotalControlSamples and TotalTreatedSamples; DEGMax = maximum number of differentially expressed genes found after removing random samples (number of samples removed on each itineration is equivalent to the corresponding number in TotalOutliers) from TotalControlSamples and TotalTreatedSamples; DEGMean = average number of differentially expressed genes found after removing random samples (number of samples removed on each itineration is equivalent to the corresponding number in TotalOutliers) from TotalControlSamples and TotalTreatedSamples; AdjPcut = Adjusted P-value cutoff used on the differential expression analysis.
