# Supplementary material for: Assessing the Impact of Sample Heterogeneity on Transcriptome Analysis of Human Diseases Using MDP Webtool
Source: Front Genet. 2019 Oct 24;10:971. doi: 10.3389/fgene.2019.00971 (PMC6822058; doi:10.3389/fgene.2019.00971)
Supplement: Figure S1 — Sample MDP scores of different human diseases. (A) Sample MDP scores of patients acutely infected with either virus or bacteria. Data were obtained from blood leukocytes and are available under GEO accession GSE6269. Healthy subjects (blue) were used as reference group. (B) Sample MDP scores of different types of cancer. Data were obtained from platelets and are available under GEO accession GSE68086. Healthy subjects (blue) were used as reference group. (C) Sample MDP scores of patients with inflammatory diseases. Data were obtained from whole blood and are available under GEO accession GSE112057. Healthy subjects (blue) were used as reference group. [file Image_1.pdf]

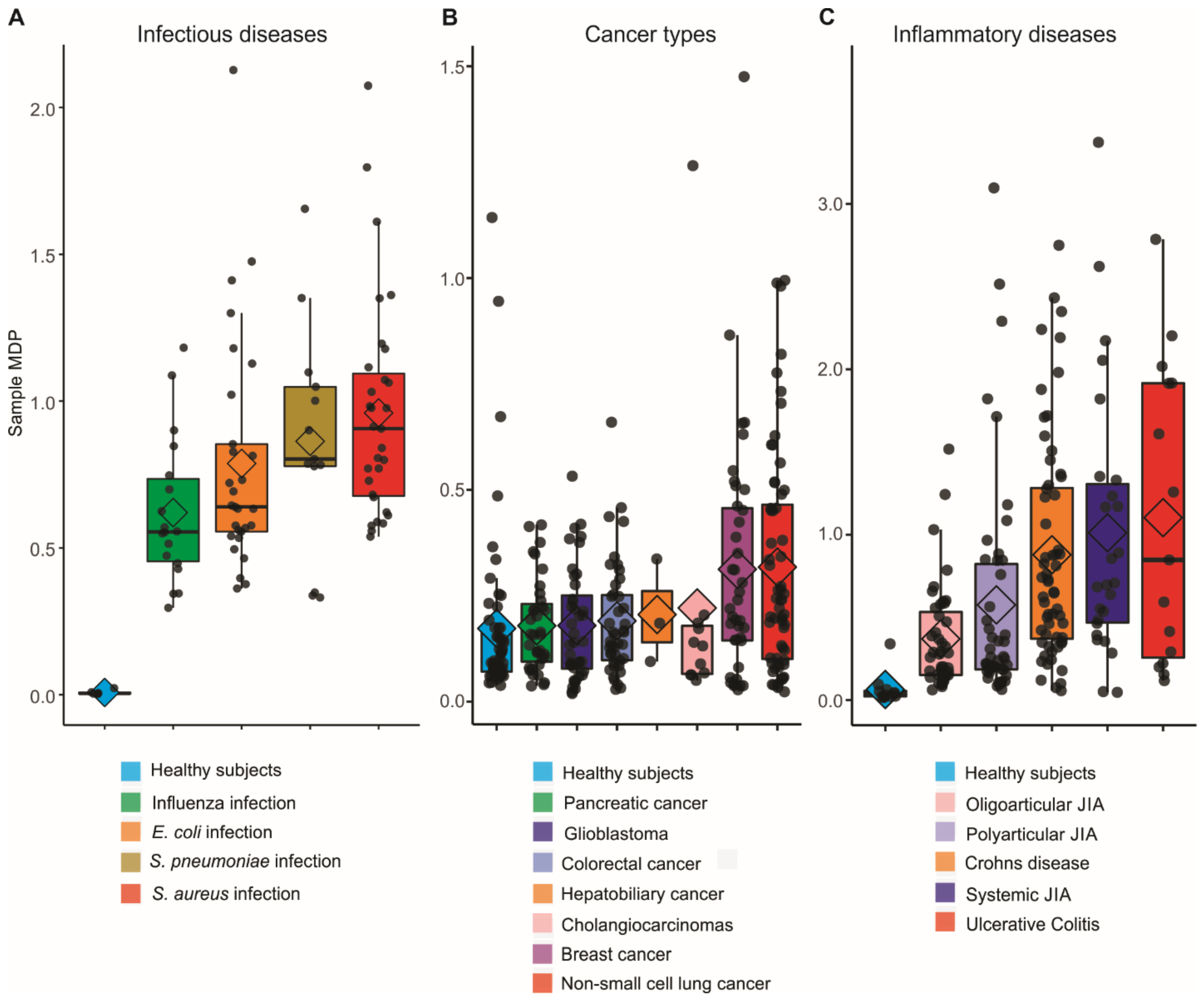

Figure S1. Sample MDP score of different human diseases. A) Sample MDP score of patients acutely infected with either virus or bacteria. Data were obtained from blood leukocytes and is available under the GEO accession GSE6269. Healthy subjects (blue) were used as reference group. B) Sample MDP score of different types of cancer. Data were obtained from platelets and is available under the GEO accession GSE68086. Healthy subjects (blue) were used as reference group. C) Sample MDP score of patients with inflammatory diseases. Data were obtained from whole blood and is available under the GEO accession GSE112057. Healthy subjects (blue) were used as reference group.
