# Supplementary material for: Assessing the Impact of Sample Heterogeneity on Transcriptome Analysis of Human Diseases Using MDP Webtool
Source: Front Genet. 2019 Oct 24;10:971. doi: 10.3389/fgene.2019.00971 (PMC6822058; doi:10.3389/fgene.2019.00971)
Supplement: Figure S2 — MDP calculated with specific gene modules. (A) Sample MDP score of patients with active TB (brown bars) and healthy controls (blue bars) using three different specific gene modules. Data were obtained from whole blood and are available under GEO accession GSE19435. (B) Sample MDP score calculated using all gene modules and for all TB datasets. The circles represent the difference between the median sample MDP score of patients with active TB and the healthy controls with no active TB within each study. The size and color of the circles are proportional to this difference. MФ: macrophages. [file Image_2.pdf]

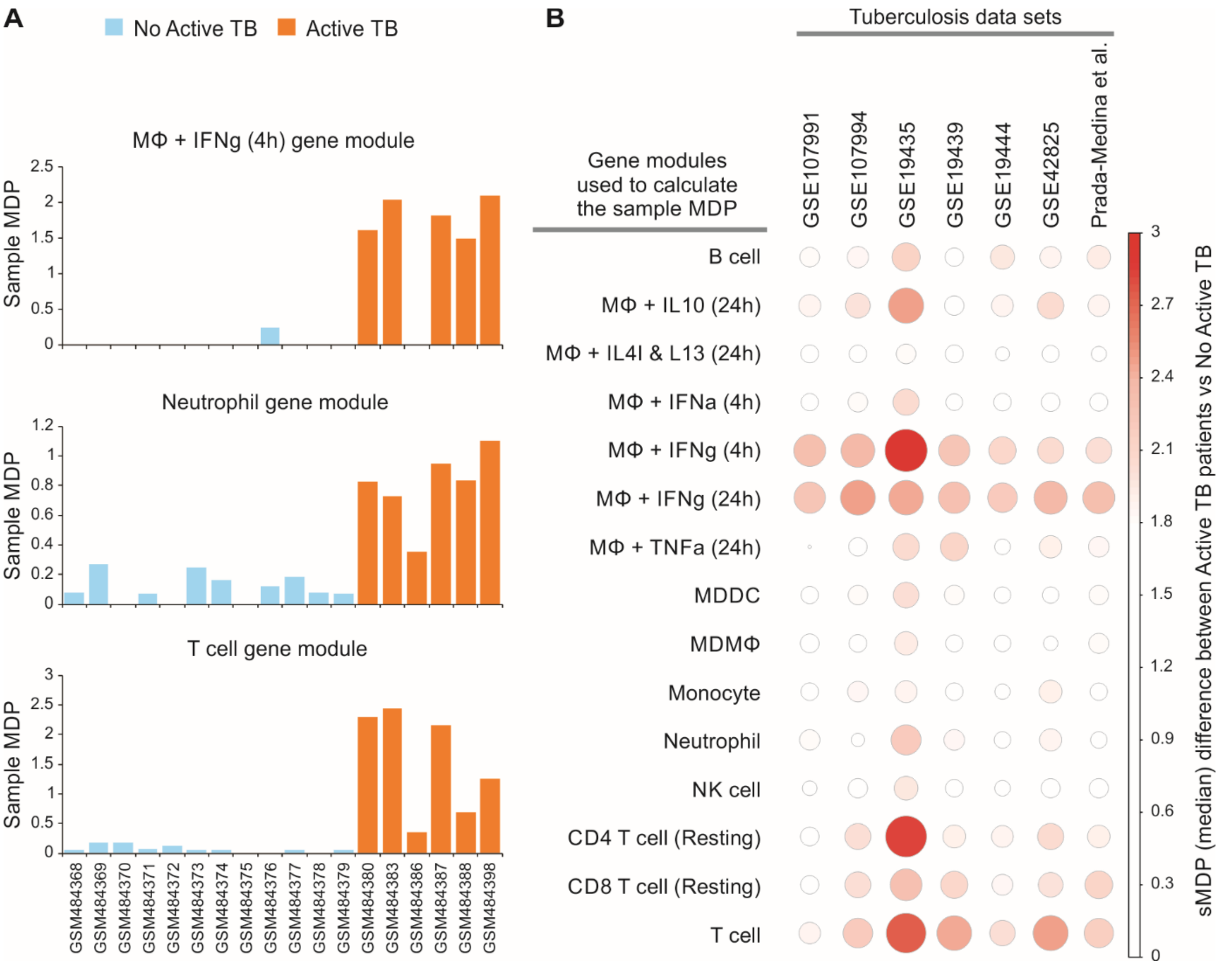

Figure S2. MDP calculated with specific gene modules. A) Sample MDP score of patients with Active TB (brown bars) and healthy controls (blue bars) using 3 different specific gene modules. Data were obtained from whole blood and is available under the GEO accession GSE19435. B) Sample MDP score calculated using all gene modules and for all TB datasets. The circles represent the difference between the median sample MDP score of patients with active TB and the healthy controls with no active TB within each study. The size and color of the circles are proportional to this difference. MΦ: macrophages.
